# Biomass-Derived Carbon Dots and Their Sensing Applications

**DOI:** 10.3390/nano12244473

**Published:** 2022-12-17

**Authors:** Jiang Fan, Lei Kang, Xu Cheng, Di Liu, Sufeng Zhang

**Affiliations:** 1 Department of Chemical Engineering, Textile and Clothing, Shaanxi Polytechnic Institute, Xianyang 712000, China; 2 School of Surveying & Testing, Shaanxi Railway Institute, Weinan 714000, China; 3 School of Chemistry and Chemical Engineering, Guangzhou University, Guangzhou 510006, China; 4 Shaanxi Provincial Key Laboratory of Papermaking Technology and Specialty Paper Development, Shaanxi University of Science and Technology, Xi’an 710021, China

**Keywords:** biomass, carbon dots, sensing, applications

## Abstract

Carbon dots (CDs) can be widely used in the field of sensing because of its good water solubility, low toxicity, high fluorescence stability and excellent biocompatibility. It has become a popular trend to prepare high-value, inexpensive, renewable and environmentally friendly CDs sensors from biomass resources. This article reviewed the research progress of biomass-derived CDs as chemical, physical and biological sensors in recent years and studied their preparation processes and sensing abilities. Furthermore, the prospects and challenges of biomass-CDs sensors were discussed. This article is expected to provide inspirations for the design, preparation and application of biomass-CDs sensors in the future.

## 1. Introduction

A novel type of fluorescent carbon nanomaterial carbon dots (CDs) have rapidly attracted research interest worldwide as the most important members of carbon nanomaterials family since its discovery in 2004 [1]. The CDs is zero-dimensional monodisperse spherical nanoparticles with an average size of less than 10 nm, which is composed of a carbogenic core with various functional groups attached to the carbon surface, such as hydroxyl, amino and carboxyl groups and therefore exhibits excellent solubility in water. They have been widely used in biological imaging, solar cells, drug delivery, catalysis and sensing applications because of low toxicity, good biocompatibility, high fluorescence stability, excellent water-solubility, adjustable fluorescence emission and excitation [2]. The synthesis strategies of CDs can be classified into two main techniques: top-down approaches (cutting larger carbon material into nanoparticles) and bottom-up methods (fusing smaller precursor molecules). Top-down approaches include oxidizing or reducing cutting, physical grinding, grinding combined with cutting, etc., but are usually faced with harsh experimental conditions, complicated operating procedures and expensive equipment, which greatly limit their practical applications. Bottom-up methods are mainly microwave-assisted method, pyrolysis, solvothermal, ultrasonic, etc. characterized by high quantum yield, cost-effective synthetic process, as well as easy and eco-friendly operation, and thus have been extensively used for CDs synthesis [3]. Many of existing researches focus on the properties and applications of CDs, but often ignore the importance of carbon sources. There are a variety of carbon sources among which renewable and environmentally friendly biomass has been extensively studied by scientists due to its low price and wide distribution. Biomass refers to all organisms produced through photosynthesis, including plant and animal derivatives, as well as industrial organic wastes [4]. Plants are known to contain sugars, vitamins, amino acids, pectin, cellulose, hemicellulose, starch, etc. Compared to plant derivatives, animal derivatives are richer in amino acids, proteins and trace elements, but not as many types and quantities as plant derivatives as reported. Industrial organic wastes are a wide range of biomass resources, including waste residues of food processing, brewing, textile and other industries. At present, biomass-derived CDs are faced with problems, such as weak luminescence intensity and low quantum yield, which can be solved by three main common routes, namely, element doping, surface modification and composites synthesis. New energy levels can be formed and initial band gaps can be adjusted by doping heteroatoms (e.g., N, S, B, P, Al, Ca, Mn, Eu and Cd), thereby regulating the luminescence and physicochemical properties of biomass CDs [5]. However, metallic element doping may have adverse effects on the biological applications of biomass-derived CDs. In addition, their fluorescence properties can be improved by combining Ag/Au nanoparticles, molecularly imprinted polymers (MIPs), polyvinylidene fluoride (PVDF), metal oxides and enzyme [6,7,8,9,10]. Chemical components (e.g., metal cations, non-metal ions, pH, gas, vapor, pesticides and organic compounds), physical factors (e.g., temperature) and biological compositions (e.g., glucose, glutathione, cytochrome c, pathogens and bacteria) all have important impacts on human safety and health of animals and plants, so it is of great significance to develop novel biomass-based CDs as sensors for their efficient detection. To the best of our knowledge, there are a few reviews on the sensing applications of biomass-based CDs. This article focuses on the recent reports (2018–2022) of biomass-based CDs as chemical, physical and biological sensors over the past five years and discusses their challenges and future development prospects, aiming to further promote their sensing applications.

## 2. Chemical Sensors

### 2.1. Metal Cation Sensors

The excessive intake of heavy metals in human bodies will pose a serious threat to health due to their non-degradability and biocumulative effect. However, traditional instrument technologies are expensive, time-consuming and professional. Therefore, it is very important to develop new biomass-based CDs sensors to efficiently recognize heavy metals.

Excessive Fe^3+^ in the natural environment has become a kind of metal ions threatening ecological environment and human health because of industrialization and anthropogenic processes [11], as it leads to Alzheimer’s and other neurogenerative diseases among the elderly [12]. Therefore, it is of great importance to achieve the selective and sensitive determination of iron. Xu Changyan et al. synthesized spherical CCDs with coconut petiole residues as the starting material through a simple hydrothermal method [13]. Based on the interaction between Fe^3+^ and carboxyl, hydroxyl or amino groups on the surface of CCDs, the sensor exhibited a wide linear range of 0.005 to 0.2 mM with a detection limit of (LOD) of 2.3 μM towards Fe^3+^ ions. According to the UV-vis absorption spectra and fluorescence decay curves, the fluorescence quenching mechanism between CCDs and Fe^3+^ was mainly explained as the synergetic effect of both static and dynamic quenching between CCDs and Fe^3+^. More importantly, relative standard deviations (2.10–13.80%) and recoveries (99.00–112.00%) in real water samples covering tap and lake water suggested that the CCDs fluorescence probe with good precision and practicability could be applied to monitor Fe^3+^ in the future. Ge Jia et al. successfully obtained N, S, P co-doped carbon dots (NSP-CDs) using green pepper seeds as precursors by employing hydrothermal method [14], which could serve as a highly selective and sensitive probe for detecting Fe^3+^ in the range of 1–500 μM with an LOD of 0.1 μM. The results of fluorescence lifetime, infrared spectroscopy and zeta potential implied that the negative phosphorus group of the NSP-CDs was statically quenched under the action of Fe^3+^. After the addition of 100 μM Fe^3+^, the intracellular fluorescence of HeLa cells decreased, suggesting that NSP-CDs could be applied to cell imaging and detection of intracellular Fe^3+^. The existing reports on CDs mostly focus on the detection of heavy metals under natural conditions. However, the PL response of CDs is unstable in acidic environment, which limits their applications in the fields of environmental pollution and metal ion sensing. Mohammed Abdullah Issa et al. prepared a highly fluorescent nitrogen-doped carbon dots (N-CDs) with carboxymethylcellulose as carbon source and linear polyethyleneimine (LPEI) as surface passivation agent/N-doping source, which could be used as a Fe^3+^ nanoprobe [15]. Under the optimal conditions of temperature (260 °C), 2-h duration and LPEI weight (1%), the quantum yield of N-CDs was as high as 44%. Surprisingly, the prepared N-CDs still emitted bright and stable radiation even after being stored at room temperature for one year. Moreover, there was a good linear relationship in the concentration range of 1 to 400 μM with a limit of detection of approximately 0.14 μM at pH of 3. Because of their high solubility, great photo-stability and intense PL capacity, N-CDs could be further used as invisible ink for data encryption and fingerprint formation. These results indicated that the sensitivity of the CDs to Fe ions could be improved by introducing N, S, P and other elements. Therefore, the selection of an appropriate precursor or dopant in the CDs fabrication plays a critical role in improving the selectivity and sensitivity toward Fe^3+^ detection, as well as chemical stability in different pH environments.

Excessive Cu^2+^ in human bodies can cause a series of diseases, including Menkes, prion, Wilson’s and Alzheimer’s [16]. Zurina Z. Abidin et al. fabricated N-doped carbon dots (N-CDs) for Cu^2+^ detection with the carboxymethylcellulose (CMC) from oil palm empty fruit bunches and linear-structured polyethyleneimines (LPEI) through hydrothermal preparation [17]. The sensing mechanism could be explained as the particularly high thermodynamic affinity of Cu^2+^ with N-chelate groups on the surface of N-CDs and rapid metal-to-ligand binding kinetics. This sensor had successfully measured Cu^2+^ in a wide linear range of 1 to 30 μM with an LOD of 0.93 μM. Sun Runcang et al. reported the exceptionally high yield (99.0%) of green CDs from biomass hydrothermal carbons treated with low-concentration sodium hydroxide solution and oxygen [18]. The reaction temperature could easily adjust the quantum yield of CDs, which was determined by the surface states and conjugated structure of the carbon core. Based on the fluorescence quenching mechanism between Cu^2+^ and CDs, the sensor exhibited a lower LOD of 85 nM in the linear concentration range of 0 to 30 μM. The novel method is also applicable to various biomass materials, including hemicelluloses, lignin, cellulose and chitosan. The low-concentration sodium hydroxide solution and oxygen were introduced into the synthetic methods of CDs, which greatly improved the yield of CDs and was conducive to the large-scale preparation of CDs in the future.

Hg^2+^ is easy to bioaccumulate in human bodies and is likely to seriously jeopardize the central nervous and endocrine systems [19]. The completely water-soluble N-CDs was synthesized by Han Aixia et al. with Highland barley as the biomass carbon source and ethanediamine as the nitrogen source through the green hydrothermal method [20]. The TEM and XRD results showed that N-CDs were the graphitic amorphous structure with narrow diameter distribution (4.5–7 nm). Due to the strong chelating ability of carboxylic groups on the N-CDs surface towards Hg^2+^, the static quenching of N-CDs was caused by the formation of non-fluorescent complex, which facilitated non-radiative electron/hole recombination annihilation. In addition, Hg^2+^ in the range of 10–160 μM was detected with a detection limit of 0.48 μM. Highland barley is a kind of abundant and cheap biomass (5 RMB/500 g in Qinghai Province, China), which contributes to the large-scale preparation of N-CDs. An ascorbic acid (AA)-enhanced CDs with Maojian of a famous green tea as carbon material was fabricated by She Yuanbin et al. [21]. The fabricated ascorbic acid (AA)-enhanced CDs presented a wide range of 0.2–60 μM and a sensitive LOD of 6.32 nM towards Hg^2+^, which provided a convenient technique for Hg^2+^ sensing in waste water, tea and rice and a great potential for Hg^2+^ determination in environmental monitoring, herbs quality and food safety. Therefore, ascorbic acid could greatly improve the detection performances of CDs on Hg^2+^. Although AA-CDs sensor was highly sensitive to Hg^2+^, Maojian was costly and not favorable for large-scale preparation in the future.

**Figure 1:**
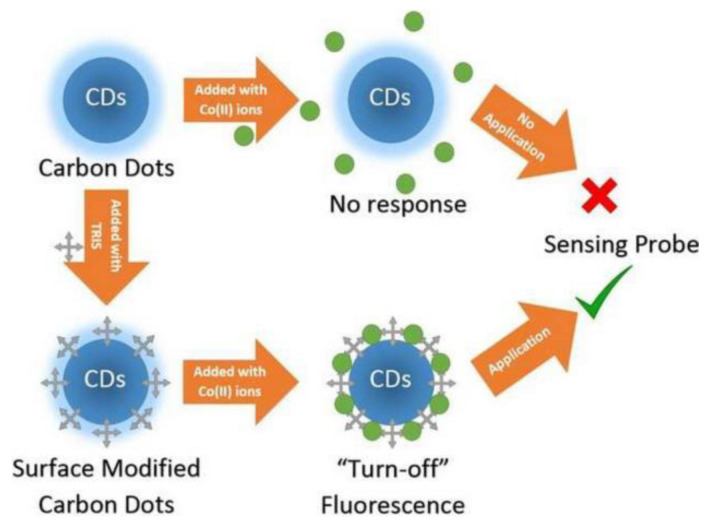
Schematic illustration of synthesis process of CDs and their application to Co^2+^ detection. Adapted with permission from Ng et al., 2018, copyright 2018. Reproduced with permission from Elsevier [23].

Excessive Co^2+^ is closely related to many diseases, including cardiomyopathy and diseases of the hearing, haematological, thyroid or cardiovascular systems [22]. Sing Muk Ng et al. reported the first CDs sensor for Co^2+^ determination using α-cellulose as the starting material and tris(hydroxymethy)aminomethane (TRIS) as capping agent by means of green thermal carbonization [23]. The results demonstrated that TRIS enhanced the specificity and sensitivity of CDs for Co^2+^ sensing (Figure 1). There was a linear fitting over Co^2+^ concentrations (16.84 μM to 1.87 mM), accompanied by a low LOD of 16.84 μM. A good overlapping between the emission spectrum of the CDs and the absorption spectrum of the Co^2+^ ions indicated the promotion of energy transfer, which was clearly proved by the experiments on the fluorescence quenching with increased concentrations of Co^2+^ with CDs. The surface functionalization of CDs facilitated the selectivity and sensitivity of CDs towards metal ions, demonstrating that the photo-chemical properties of CDs could be altered by subsequent post-synthesis treatment, such as surface passivation and hybridization into nanocomposites.

Cr^6+^ (Cr_2_O_7_^2−^) is more easily absorbed by human bodies than Cr^3+^, and its toxicity can corrode skin and thus potentially lead to vomiting, cramps, convulsions and even death [24]. Qiu Fengxian et al. designed novel chitosan-modified CDs (CDs/CTS) connected together by the physical interaction of intermolecular hydrogen bonds and the chemical interaction between hydroxyl and amino units in chitosan (CTS) and hydroxyl groups in CDs [25]. The FTIR, XPS and TEM results proved that CDs/CTS had a spherical outline and a uniform size distribution (1.0–3.5 nm). The fluorescence emission of CDs/CTS at 441 nm was quenched by Cr_2_O_7_^2−^, which could be interpreted as the photoluminescence-inactivation effect induced by higher binding affinity and faster chelating kinetics of N/O groups on the CDs/CTS surface with Cr_2_O_7_^2−^ than other metal ions. Meanwhile, a linear equation of Cr_2_O_7_^2−^ concentrations (0–600 μM) and LOD (0.72 mg/L) was obtained. Relative standard deviations (0.54–0.84%) and recoveries (93.52–97.47%) in soil suggested that the fluorescence CDs/CTS probe with good precision and practicability could be employed to monitor Cr_2_O_7_^2−^ in real environments. However, because CDs/CTS was only suitable for neutral or alkaline medium, it would dissolve in an acidic environment, leading to a rapid decline in its fluorescence intensity.

Residual Mn^7+^ (MnO_4_^−^) in drinking water causes discoloration and a metallic taste, and thus skin irritation and gastrointestinal distress [26]. Wun Jern Ng et al. designed one-step hydrothermal carbonization N/Al-CDs using durian shell as the main carbon source and urea and aluminum nitrate as dopants [27]. The results showed that the quantum yield of N/Al-CDs reached 28.7%, higher than that of N-CDs (10.4%), indicating that the luminescence properties of N-CDs were improved by introducing an Al element in N-CDs. More importantly, the N/Al-CDs sensor presented an outstanding selectivity for MnO_4_^−^ (0–100 μM) with an ultralow LOD of 46.8 nM. N/Al-CDs could be used to selectively detect MnO_4_^−^, which was explained as the transfer process of non-radiative light induced electron transfer (PET) oxidation from purple MnO_4_^−^ to brown Mn^4+^. The N/Al-CDs provided a great potential for MnO_4_^−^ recognition in real water samples (e.g., lake water and tap water). The introduction of Al element doping in the preparation process of CDs adjusted the electronic transition and electronic mobility and improved the optical and physical-chemical properties of CDs, but the presence of aluminum made biological applications not achievable.

**Figure 2:**
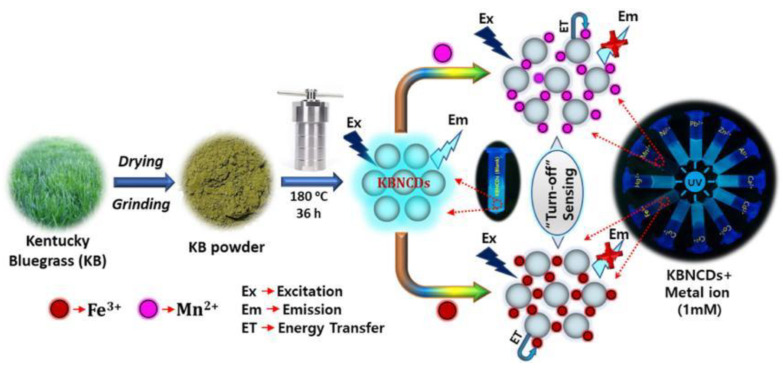
Schematic representation of the preparation process of KBNCDs and a possible quenching mechanism. Adapted with permission from Krishnaiah et al., 2019, copyright 2019. Reproduced with permission from Elsevier [29].

Due to the high stability of Mn^2+^ in the ecosystem, an excessive intake by human bodies can cause several diseases, such as neurological disorders [28]. Byong-Hun Jeon et al. designed strong cyan-blue KBNCDs by one-step hydrothermal treatment using waste biomass Poa Pratensis (Kentucky bluegrass, KB) as carbon material and ethylenediamine as nitrogen source [29]. The X-ray photoelectron spectroscopy (XPS) and Fourier transform infrared spectroscopy (FTIR) results demonstrated that abundant hydroxyl, carboxyl and amine units on the KBNCDs surface was responsible for an excellent opportunity for the complexation of KBNCDs with Mn^2+^ or Fe^3+^. The concentrations of KBNCDs and Mn^2+^ or Fe^3+^ ranged from 5.0 to 25 µM and the LOD for Mn^2+^ and Fe^3+^ was calculated as 1.2 µM and 1.4 µM, respectively. The schematic representation of the preparation process of KBNCDs and a possible quenching mechanism are shown in Figure 2. The complex formation between KBNCDs and Mn^2+^ or Fe^3+^ led to charge transfer and non-radiative recombination, leading to a decline in fluorescence intensity. So far, KBNCDs can not distinguish Mn^2+^ from Fe^3+^ although a few biomass-derived carbon dots can be simultaneously used to recognize Mn^2+^ and Fe^3+^.

Though Ag^+^ can be widely applied in medical, skin care, pharmaceutical, electrical and other fields, excessive Ag^+^ can be harmful to both human bodies and the ecological environment [30]. Using bean pods (rich in nitrogen units) and onions (rich in thiol groups), Xu Shoufang et al. prepared a novel colorimetric S, N/CDs sensor for Ag^+^ sensing by hydrothermal technology [31]. After the addition of Ag^+^ with a concentration of 0.5 µM, the S, N/CDs sensor achieved good visual detection, and the color of the mixed solution was changed from light yellow to deeper brown under sunlight. The fluorescence intensity of the mixed solution of S, N/CDs and Ag^+^ decreased significantly, which was potentially attributed to aggregation induced quenching. With the addition of Ag^+^ overnight, a black precipitate was clearly observed at the bottom of the test bottle, which was caused by the aggregation of large particles according to TEM results. Over a wide working concentration range of 0.1–25 µM, the S, N/CDs sensor exhibited higher selectivity for Ag^+^ than other metal ions, with an LOD of up to 37 nM, lower than that at the minimum concentration of Ag^+^ standardized by the World Health Organization (WHO). When 4T1 cells were immersed in 200 µg/mL S, N/CDs solution for 24 h, strong blue fluorescence appeared in 4T1 cells. Pods and onions are inexpensive and have great potential for environmental monitoring, intracellular Ag^+^ detection and cell imaging. Therefore, heteroatom-rich biomass was selected as initial carbon source due to its capability to improve sensing performance. Compared with foreign nitrogen and sulfur chemicals, self-doped biomass is more environmentally friendly and more in line with the government’s “zero carbon emission” strategy.

Cd^2+^ can cause various diseases, such as high blood pressure, nervous system and kidney [32]. Wang Yujuan et al. developed the N-doped CQDs with Auricularia auricular and ethylenediamine as precursors via one-step hydrothermal process [33]. The absolute value of zeta potential of N-CQDs was 25.2 mV, which proved their better physical stability. With the addition of Cd^2+^ or Hg^2+^, the fluorescence intensity of N-CQDs was effectively quenched as a result of static quenching. Moreover, a good linear relationship occurred in the concentrations of 0 to 50 μM, and LOD for Cd^2+^ and Hg^2+^ was approximately 101.55 nM and 77.21 nM, respectively. Relative standard deviations (2.35–4.23%) and recoveries (80.0–107.7%) in actual samples suggested that fluorescence N-CQDs probe could be used to analyze Cd^2+^ and Hg^2+^ in dendrobium plant. Although the N-CQDs still failed to separate Cd^2+^ from Hg^2+^ though it was able to simultaneously detect them.

The accumulated Ru^3+^ in human bodies will cause serious damages to skin and eyes [34]. However, there are still few fluorescent sensors that can effectively detect Ru^3+^. Lin Xu et al. synthesized a CDs sensor to detect Ru^3+^ using starch as a precursor material [35]. With the increase of temperature, the particle size of CDs first increased and then decreased, which was closely related to the quantum yield of CDs. Thus, temperature had a great effect on the size of CDs. When temperature was 300 °C, the quantum yield of CDs was the largest (21%). No blue fluorescence was observed when the pyrolysis temperature of starch was lower than 250 °C or higher than 350 °C^.^ The fluorescence emission intensity of CDs was the highest at pyrolysis temperature of 300 °C. The fluorescence intensity of CDs decreased significantly in the presence of Ru^3+^, while changing little in the presence of other ions, indicating that the CDs had a good selectivity towards Ru^3+^ due to a stronger coordination between CDs and Ru^3+^. Due to its low price of starch biomass sources (potatoes, sweet potatoes and corn, etc.), it is very meaningful to prepare high-value sensors for Ru^3+^ detection. The reaction temperatures used for CDs preparation were lower than 220 °C and that used in this article was 300 °C, which was a big challenge for equipment safety.

### 2.2. Non-Metal Ion Sensors

Highly toxic fluoride ion is a major pollutant in groundwater and its excessive amount can cause dental and skeletal fluorosis [36]. However, traditional methods used for fluoride ion detection are faced with various limitations, such as a trained workforce, fully-equipped laboratory and complex instrumentation, etc. Therefore, it is of great significance to develop a novel biomass-based CDs sensor for fluoride ion sensing. An on-off-on fluorescence nanoprobe CQDs-Eu^3+^ using taro peels waste via ultrasonic-assisted wet-chemical-oxidation process and Eu^3+^ was developed by Binoy K Saikia et al., which realized fluoride ions sensing in water [37]. The combination of COOH units on the CQDs surface and Eu^3+^ led to a faster fluorescence quenching of CQDs, which was proved by the formation of some micro-rods-like structures of CQDs-Eu^3+^ nanoprobe according to the Field Emission-Scanning Electron Microscope (FE-SEM) and elemental mapping analysis results. More interestingly, Eu^3+^ had a great affinity for fluoride ions, which resulted in the regeneration of fluorescence emission of CQDs and the disintegration of micro-rods-like structures. Unfortunately, the fluorescence intensity of the mixed solution was not linearly associated with the concentrations of fluoride ions. The CQDs synthesized using the ultrasound-assisted wet-chemical-oxidation process was more gentle than those prepared by hydrothermal method.

**Figure 3:**
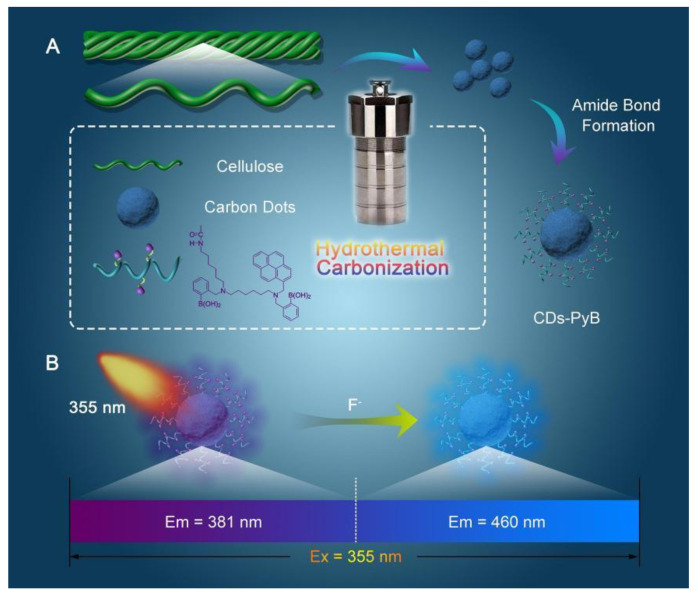
Schematic illustration of the synthetic process of CDs-PyB and the procedure of CDs-PyB. for fluoride ions detection. Adapted with permission from Li et al., 2020, copyright 2020. Reproduced with permission from Elsevier [39].

Boric acid can be used to prepare biomase-based CDs because of its unique binding properties to fluoride and its inherent selectivity to other anions [38]. Li Meng et al. constructed a pyrene-boronic acid-based CDs probe (CDs-PyB) to detect fluoride ions utilizing PyB groups immobilized on the surface of CDs derived from carboxymethylated nanocellulose (C-CNC) [39]. The schematic illustration of the synthetic process of CDs-PyB and the detection procedure of CDs-PyB for fluoride ions are shown in Figure 3. Moreover, the CDs-PyB sensor exhibited good sensitivity with an LOD of 59 μM towards fluoride ions over a wide range of 0–200 μM. Meanwhile, an amino-functionalized cellulose membrane containing CDs-PyB presented strong fluoride ions sensing ability, removal efficiency (90.2%) and excellent cytocompatibility. However, the interference effect of CDs-PyB on the detection process of fluoride ions in the presence of more other anions remains to be further explored.

**Figure 4:**
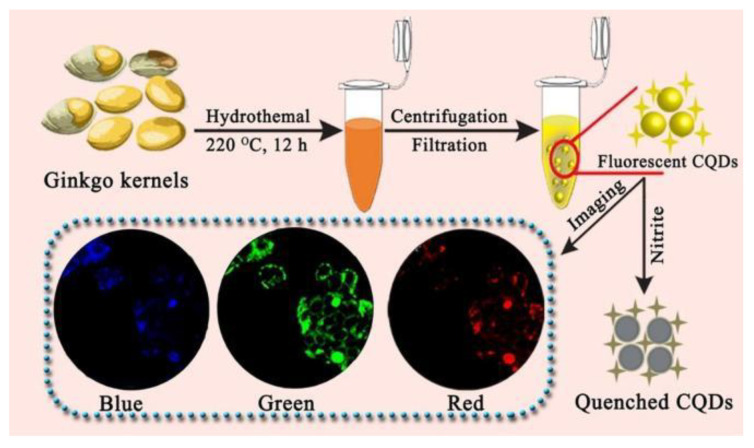
Schematic illustration of the preparation process of CQDs and its determination of nitrite ions and cell imaging. Adapted with permission from Zhang et al., 2022. Copyright 2022. Reproduced with permission from Elsevier [41].

Excessive consumption of nitrite ions has been linked to an increased risk of cancer and hypertension, but few fluorescent sensors for nitrite ions sensing have been reported [40]. Using ginkgo kernel as raw material, Zhang Qianchun et al. prepared a carbon quantum dots (CQDs) with a quantum yield of 37.8% to recognize trace nitrite ions [41]. After the addition of nitrite ions, the fluorescence intensity of CQDs was significantly quenched, which was potentially attributed to the interaction between nitrite ions and CQDs, leading to a photoinduced electron transfer effect. An LOD of 0.15 μM was achieved, accompanied by a linear relationship between fluorescence response and the concentration of nitrite ions (0.50–50 μM). The determination results of nitrite ion concentration in corn sausage, ham sausage, mustard and hot dog samples by the CQDs sensor were consistent with HPLC-UV technology, indicating that the CQDs were accurate and suitable for the detection of nitrite ion in real samples. In addition, CQDs exhibited lower cytotoxicity and better biocompatibility. When MCF7 cells were incubated in CQDs solution for 24 h, fluorescence of different colors was observed under different excitation wavelengths. However, green fluorescence images were more legible and specific than those that were blue and red. The preparation process of CQDs and its determination of nitrite ions and cell imaging are shown in Figure 4. The ginkgo kernel-based CQDs provided a new method for nitrite ions detection and cell imaging.

Borax is widely used in industrial and commercial products, but it will cause various symptoms, such as vomiting, fatigue, proliferation of immune cells and kidney diseases, if excessive amount is accumulated in human bodies [42]. Peerasak Paoprasert et al. reported acarbon dots (CDs) for borax sensing prepared by water hyacinth leaves via the acid-treatment-assisted pyrolysis method [43]. The fluorescence intensity of CDs was quenched by borax, resulting from charge transfer between CDs and borax through their favorable lowest unoccupied molecular orbitals according to density functional theory (DFT) and time-dependent density functional theory (TD-DFT). The LOD was estimated to be 1.5 μM. At the same time, a portable article-based device for the on-site detection of borax was developed with an LOD of 11.85 μM. To the best of our knowledge, the use of biomass-based CDs sensors for borax detection has been reported for the first time. The fluorescence quantum yield of CDs prepared by two-step synthesis method was as high as 27.0%, while that of CDs obtained by one-step approach without acid treatment was only 6.5%, indicating that the quantum yield of CDs could be improved by nitric acid treatment.

### 2.3. Gas, Solvents and Vapor Sensors

**Figure 5:**
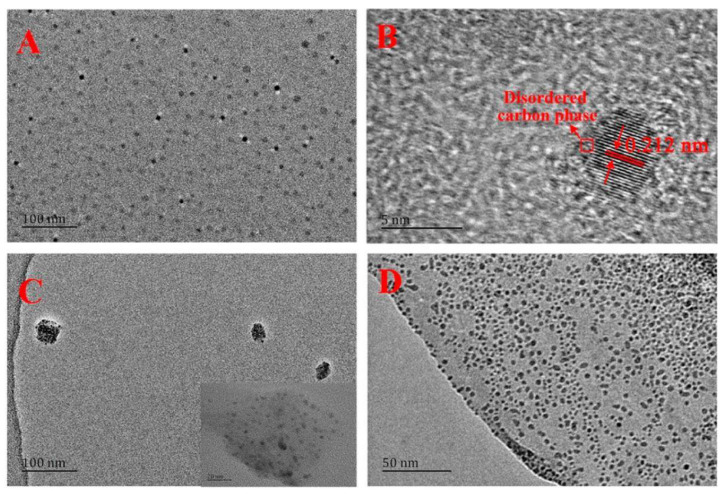
HRTEM image of BD-CDs (**A**,**B**) in ethanol, (**C**) after adding Al^3+^ in ethanol, (**D**) in water. Adapted with permission from Rao et al., 2020, copyright 2020. Reproduced with permission from American Chemical Society [45].

In general, water is not toxic, but it is still critical to accurately and efficiently detect water food processing, pharmaceutical manufacturing and biomedical monitoring [44]. Liu Tao et al. reported a biomass-based carbon dots (BD-CDs) for simultaneously detecting H_2_O and Al^3+^, which was synthesized from leaves of cinnamomum plants via solvothermal treatment [45]. In the presence of H_2_O, the fluorescence intensity of BD-CDs at 470 nm remained unchanged, while that at 670 nm decreased significantly. The TEM results indicated that there was no obvious aggregation of BD-CDs after the addition of water, which was due to the solvent effect of hydrophobic structure of the chlorophyll-derived porphyrins. Moreover, based on the linear equations between the fluorescence intensity of BD-CDs and H_2_O contents (0.5–30% and 30–70%), BD-CDs demonstrated high sensitivity with an LOD of 0.022%. After the addition of Al^3+^, the fluorescence emission of BD-CDs at 470 nm was intensively enhanced while that at 670 nm remained basically unchanged as a result of aggregation-induced emission enhancement (AIEE). BD-CDs added with water agglomerated into nanospheres with dimension distribution (30–70 nm) (Figure 5). There was a good linearity in the Al^3+^ concentration range of 0.3–100 nM and a low LOD of 0.25 nM. More interestingly, smartphone-based BD-CDs platforms had a wide range of application scenarios in the fields of food safety and environmental monitoring. At present, water is the major medium for CDs detection, while ethanol was used for testing in this article, which would potentially pollute the environment in the actual sample applications.

**Figure 6:**
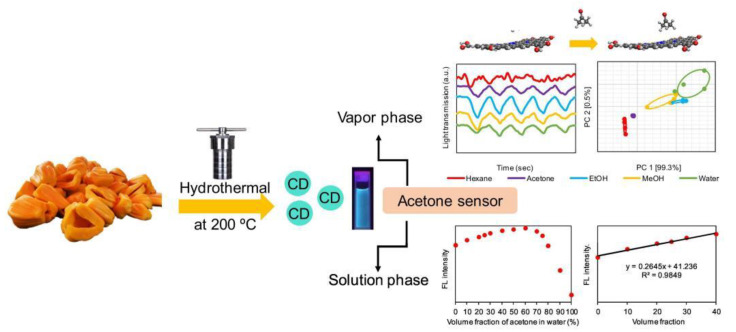
Schematic illustration of the synthesis and application of CDs from jackfruit. Adapted with permission from Thongsai et al., 2019, copyright 2019. Reproduced with permission from Elsevier [47].

Acetone is a widely used solvent, but it is harmful, toxic and dangerous with low vapor pressure. When exposed to high levels of acetone, human bodies will suffer a lot of illnesses, including dermatitis, irritation, headaches, fatigue, nausea, etc. [46]. Peerasak Paoprasert et al. fabricated carbon dots (CDs) derived from jackfruit for acetone sensing in aqueous solution by the hydrothermal method [47]. The fluorescence intensity of CDs gradually increased with the increase of acetone concentration (0–70% *v*/*v*), but decreased when acetone concentration exceeded 70% *v*/*v* due to the limited solubility of CDs in acetone. In the meantime, the CDs successfully determined acetone in water in the concentration range of 0–40% *v*/*v* with an LOD of 1.5% *v*/*v*. The synthesis and application of CDs from jackfruit is shown in Figure 6. DFT calculations confirmed that these interfacial interactions and electronic coupling caused changes in electronic states and then altered the optical properties of CDs. At the same time, the CDs films could be integrated into the optical electronic nose to detect acetone vapor in a real-time manner at room temperature. The CDs had strong electron donating and accepting properties, demonstrating their potential applications in ambipolar electronic devices. However, the quantum yield of CDs in solution was only 5.2%, which greatly limited their practical detection applications in the future.

When exposed to alcohol vapor for a long time, human bodies will suffer irritation, intoxication and even death. Peerasak Paoprasert et al. successfully detected alcohol vapors by using hydrothermal methods to extract CDs from rice husks and integrating them into a film electronic nose [48]. After incubation in the CDs solution (80 μg/mL) for 72 h, cell viability still exceeded 80%, indicating that the CDs had an excellent biocompatibility. When the film was exposed to alcohol vapor, sensor response dropped rapidly, caused by increased absorptivity and reduced transmitted light of the film. The sensor presented excellent selectivity towards alcohol vapor with a low LOD of 0.025%. The rice husk-based CDs provided a great path for bioimaging and food safety monitoring. There is abundant rice in the world, which produces sufficient rice husks and thus lays a foundation for the large-scale preparation of CDs with cheap materials.

Excessive ammonia results in the eutrophication of the water environment and damages the entire ecosystem. Long-term consumption of ammonia-containing water would pose a serious threat to human health [49]. Li Nianbing et al. prepared a smartphone-coalesced CDs nanoprobe using onion juice precursor through hydrothermal route, which could serve as a sensor for pH and ammonia [50]. There was a good linear correlation between the absorbance of CDs solution at 340 nm and broad pH values (2.0–12.0). According to the TEM results, the CDs were well dispersed in solution with a pH value of 2.0, but then flocculated into a flower-like nanocluster at a pH of 6.0. Subsequently, at a pH of 12.0, large amorphous structure emerged with the increase of intermolecular interaction of hydrogen bonds between CDs. In addition, there was a linear relationship between the fluorescence intensity of CDs solution and pH value (6.1–13.6). After the absorption of ammonia gas, CDs solution was alkalized, and the pH value increased, leading to the fluorescence quenching of CDs and achieving the aim of detecting ammonia over the ammonia concentration range of 0.1–50 mM with an LOD of 34 μM. Surprisingly, the fluorescence emission of CDs solution mostly remained unchanged after being incubated in dissolved ammonia for 7 d. This is the first time that CDs has used the headspace single drop microextraction (HS-SDME) technology to detect ammonia gas, showing great potential in the environmental field. Ammonia was prepared through the reaction between ammonium ions and alkaline solution in this article. However, ammonia had certain solubility in solution, which affected the detection performances of CDs.

H_2_O_2_ is mainly used to sterilize wounds and foods, but when inhaled in small amounts, it can induce symptoms, such as skin irritation, chest pain and breathing difficulties, etc. [51]. Yang Xiaoming et al. synthesized carbon dots (CDs) using carboxymethylcellulose as carbon precursor for simultaneously sensing isoniazid and H_2_O_2_ [52]. The fluorescence intensity of CDs at 420 nm decreased obviously after the addition of isoniazid (1 mM), but recovered immediately with the addition of H_2_O_2_ (600 nM). After being added with isoniazid, the size of the CDs changed from 6.3 ± 1.3 nm to 22.3 ± 0.8 nm, which may be caused by the combination of the CDs and isoniazid. The size of the CDs was then reduced to 10.8 ± 1.2 nm as a result of the oxidation of isoniazid by H_2_O_2_. The fluorescence detection mechanisms of CDs for isoniazid and H_2_O_2_ may be attributed to photoinduced electron transfer (PET) and the inner filter effect (IFE) according to the UV-vis absorption experiment results. The LOD was estimated to be 5.1 nM over the isoniazid concentrations ranging from 20 nM to 6 µM. Meanwhile, the linear range for discriminating H_2_O_2_ was 4–800 nM and an LOD of 1.6 nM was obtained. The RSD and extraction recovery values for isoniazid and H_2_O_2_ in urine, serum and milk samples suggested that the sensing system had a great potential in the actual application scenarios. Although the sensor CDs were able to simultaneously detect isoniazid and H_2_O_2_, it would make the operation more difficult and complex in future practical applications.

### 2.4. Pesticide Sensors

Although pesticides (e.g., dimethoate, dichlorvos, isocarbophos, mesotrione and thiram) are extensively used in plant pests and diseases, their residues contaminate crops, soil and water and the residues will cause serious illnesses if they are absorbed by human bodies. Various traditional methods, such as high-performance liquid chromatography (HPLC), gas chromatography and electrochemical sensor, can detect pesticide residues, but they are time-consuming, expensive and complicated. Therefore, it is very urgent to develop new biomass-based CDs sensors for detecting pesticide residues.

**Figure 7:**
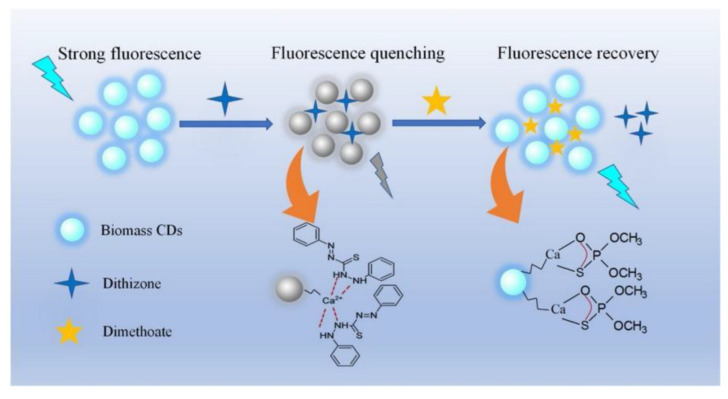
Schematic illustration of the fluorescence switch mechanism for dimethoate detection. Adapted with permission from Liu et al., 2020, copyright 2020. Reproduced with permission from Elsevier [53].

Dinglan et al. extracted animal waste biomass CDs from pig ribs (food waste) by hydrothermal method and acid carbonization method, and constructed biomass CDs for Dimethoate sensing [53]. According to the experimental data of FT-IR and XPS tests, the fluorescence properties of CDs could be improved by doping calcium, sulfur and nitrogen elements of pork rib bones. By means of coordination effect, dithizone could effectively quench the fluorescence of CDs. However, after the introduction of dimethoate, the dithizone ligands on CDs were replaced by hydrolysates, thus restoring the fluorescence of CDs by a simple displacement mechanism. Based on the test results of UV-vis absorption and fluorescence lifetime, the fluorescence quenching mechanism of CDs may be fluorescence resonance energy transfer (FRET) process, and the fluorescence intensity of CDs was restored with the addition of dimethoate, which may be related to the closure of the FRET route (Figure 7). There was a linear fitting between the intensities of CDs and dimethoate concentration (0.15–5.0 µM), together with a good LOD of 0.064 µM. The recoveries value (93–105%) and relative errors (2–6%) in farm water and river water indicated that the CDs sensor provided an accurate and reliable approach for pesticide residue (dimethoate) measurement in fruits, vegetables and water samples. The self-doping of calcium, sulfur and nitrogen elements in pork rib bones could improve the photophysical and chemical properties of CDs, while effectively avoiding the waste of resources.

Li Jinfang et al. fabricated nitrogen and sulfur co-doped carbon dots (NS-Cdots) derived from low-price leek for dichlorvos detection through acetylcholinesterase/choline oxidase-based cascade enzymatic reaction [54]. After the addition of H_2_O_2_, the fluorescence of NS-Cdots was quenched, which indicated that NS-Cdots could be used as an indicator for the biological analysis of oxidase. Acetylcholinechloride (ACh) was catalyzed into choline by acetylcholinesterase (AChE). Then, choline was further oxidized to betaine and byproduct H_2_O_2_ under the catalysis of choline oxidase (ChOx), which quenched the fluorescence of NS-Cdots. Because of the inhibition effect of dichlorvos on AChE, the addition of dichlorvos in the above system would reduce the choline yield, which generated H_2_O_2_. Therefore, the fluorescence of NS-Cdots basically remained unchanged. The linear range for discriminating dichlorvos concentration was 1 nM–1 mM and an LOD of 0.5 nM M was obtained. Recoveries (96.0–104.0%) and RSD values (lower than 3.3%) indicated that the NS-Cdots provided a feasible method for dichlorvos recognition in Chinese cabbage, a kind of primary agricultural products. After being cultured in NS-Cdots (300 μg/mL) solution for 24 h, T24 cells maintained high cell viability and could be used in biomedical field. Although the NS-Cdots sensor achieved the ultrasensitive detection of dichlorvos, the cascade enzymatic reaction testing method was relatively cumbersome and not easy for untrained technicians.

Hu Yuefang et al. realized the ultrasensitive detection of isocarbophos by using natural soybean biomass-based nitrogen-codoped carbon dots (B-NCdots) via pyrolysis and media-linked reaction [55]. The blue fluorescence of B-NCdots was quenched by Cu^2+^ due to the non-radiative electron transfer from B-NCdots to Cu^2+^. Acetylthiocholine (ATChCl) was catalyzed by acetylcholinesterase (AChE) to produce thiocholine, which then reacted with Cu^2+^ to restore the fluorescence of B-NCdots. However, the activity of acetylcholinesterase and fluorescence recovery of B-NCdots were inhibited in the presence of suprathophos to further sense suprathophos. According to the linear equation between the fluorescence intensity of B-NCdots and isocarbophos concentration (1 nM–0.1 mM), the LOD was calculated to be 0.3 nM. Based on the above method, the concentration of isocarbophos in three broccoli samples were 1.20–1.25 mg/kg, exceeding the maximum residual limit of isocarbophos in China (0.1 mg/kg). In addition, the B-NCdots indicated a great potential in living cell imaging, food safety and water environment. Although B-NCdots were very effective in detecting isocarbophos, test system required the joint participation of Cu^2+^, ATChCl and AChE, which was likely to produce interference impurities and was not very reproducible.

A fluorescence platform CQDs@MIPs for selectively recognize mesotrione was constructed by Chen Ligang et al. by combining CQDs derived from mango peels with molecularly imprinted polymers (MIPs) through hydrothermal method and sol-gel technology [56]. The integration of low-cost, easy-to-prepare and reusable MIPs with CQDs could improve the selectivity of fluorescence detection. A spherical structure of CQDs@MIPs presented a diameter distribution (30–50 nm), which was significantly larger than that of CQDs. In the presence of mesotrione, the fluorescence of CQDs@MIPs was quenched due to static quenching and electron transfer, according to the experimental results of Stern-Volmer equation, UV absorption spectra and fluorescence lifetimes. The fluorescence intensities of CQDs@MIPs was linearly associated with the concentration of mesotrione in the range of 15 nM–3 μM, with an LOD of 4.7 nM. Satisfactory results of RSDs (3.2–6.1%) and recoveries suggested that CQDs@MIPs had been successfully applied to the determination of mesotrione in corn samples. The complex MIPs technology is still not applicable to large-scale preparation and practical applications despite its ability to improve the sensing performance of CQDs.

**Figure 8:**
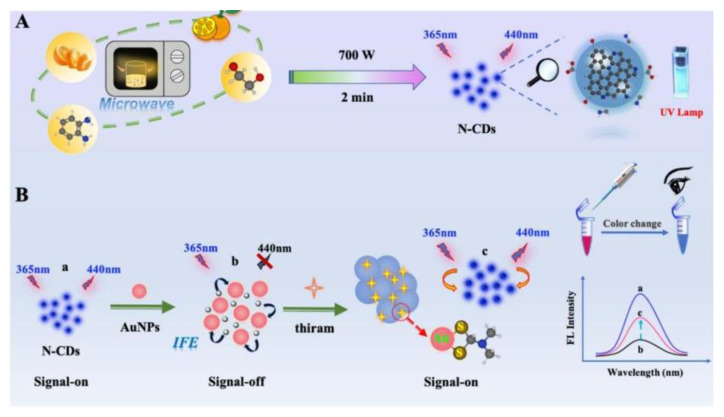
Schematic illustration of an AuNPs and N-CDs based fluorescent “on-off-on” nanosensor for thiram detection. (**A**). the preparation process of N-CDs. (**B**). the detection process of nanosensor. Adapted with permission from. Fu et al., 2022, copyright 2022. Reproduced with permission from Elsevier [57].

Kong Weijun et al. prepared an on-off-on fluorescent probe based on gold nanoparticles (AuNPs) and biomass orange peel derived nitrogen-doped carbon dots (N-CDs) to ultra-sensitively detect thiram [57]. More surprisingly, N-CDs were prepared in a microwave oven after being heated for merely 2 min. According to the UV-vis absorption spectra, the strong fluorescence emission of N-CDs was quenched due to the inner filter effect, Zeta potential and fluorescence lifetime in the presence of AuNPs. Then, after the addition of thiram to the mixed system N-CDs-AuNPs, the fluorescence intensity of N-CDs recovered with a visible color change from red to blue, which was attributed to the aggregation of AuNPs caused by the combination of thiram and AuNPs through Au-S bonds (Figure 8). A linear equation of thiram concentration (10–200 ng/mL) and fluorescence intensity of N-CDs was obtained, with an ultralow LOD of 4.7 ng/mL. The satisfactory results with recoveries (102.22–107.57%) and RSDs values (less than 5%) in hawthorn samples indicated that the sensing system N-CDs had a good application prospect for monitoring thiram in food safety and environmental pollution. The reaction performed by traditional hydrothermal method generally lasted 2 h, but it only took 2 min to prepare N-CDs, which greatly saved energy. The introduction of AuNPs not only made the sensor expensive but was also not conducive to biological applications.

### 2.5. Organic Compound Sensors

**Figure 9:**
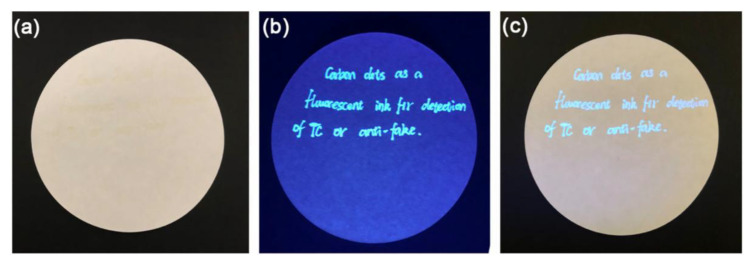
Photographic images of articles with hand-written characters using N-CDs as a fluorescent ink under day light (**a**), UV light (**b**) and both of them (**c**). Adapted with permission from. Zhao et al., 2020, Copyright 2020. Reproduced with permission from Elsevier [59].

Tetracycline analogues are widely used in veterinary and human medicine as antibiotics, but because overused, they will accumulate in food and cause serious risks to human health, such as yellow teeth, liver damage and allergic reactions [58]. The conventional methods of tetracycline detection were high-performance liquid chromatography (HPLC) and LC with tandem mass spectrometry (LC-MS), which have various disadvantages, such as professional skill, tedious operation procedure and high cost. Sun Runcang et al. designed a novel N-CDs sensor to identify tetracycline utilizing waste cellulose diacetate (CDA) from discarded cigarette filters as a carbon source and ammonium hydroxide as the passivation agent through one-pot hydrothermal carbonization [59]. Based on the experimental results of UV-vis absorption and fluorescence lifetime, the fluorescence intensity of N-CDs was obviously quenched after the addition of tetracycline, which was potentially caused by inner filter effect. The average size (1.71 nm) of N-CDs in the absence of tetracycline was basically the same as that (1.72 nm) in the presence of tetracycline, suggesting that tetracycline did not cause the aggregation of N-CDs. An LOD of 0.06 μM was achieve with a linear relationship between the quenching efficiency and tetracycline concentration (0–80 μM). The photos of hand-written characters on article exhibited bright blue fluorescence emission under ultraviolet light at 365 nm, indicating that N-CDs fluorescent ink had a potential application prospect in the fields of information storage and encryption (Figure 9). Guo Zhanhu et al. synthesized nitrogen-doped carbon quantum dots (N-CQDs) for tetracycline analogs detection by hydrothermal method with rice residue as carbon precursor and glycine working as nitrogen source [60]. According to the linear equations, the LOD values of terramycin (OTc), chlortetracycline (CTc) and tetracycline (TC) were calculated to be 0.3739 μM, 0.2791 μM and 0.2367 μM, respectively. The concentration values of TC in real water samples measured by fluorescence spectrometry were almost similar to that measured by UV-vis method, which indicated that fluorescence method was able to rapidly and accurately monitor TC concentration in practical applications. N-CQDs were not capable to distinguish OTc, CTc and TC though they could simultaneously detect them.

Ding Lan et al. fabricated a novel CDs-MIP sensor for detecting oxytetracycline with CDs (derived from sweet potato peels) coupled with molecularly imprinted polymers (MIP) through hydrothermal and sol-gel polymerization approaches [61]. The average particle sizes (31 ± 7 nm) of CDs-MIP were larger than that of CDs (2.0 ± 0.6 nm), which implied that the surface of CDs was well coated with MIP. The fluorescence intensity of CDs-MIP added with oxytetracycline was decreased, which may be caused by electron-transfer-induced fluorescence quenching mechanism. The LOD for oxytetracycline was 15.3 ng/mL, higher than that obtained by some other methods, such as HPLC-MS/MS. The CDs-MIP sensor was able to sense oxytetracycline in honey samples with RSDs (2.3–4.1%) and recoveries (90.2–97.3%). The interference of common substances could be completely eliminated by combining the predefined properties of MIP technology with the special response of ratio fluorescence, thus greatly improving the selectivity and sensitivity of target substances. Niu Na et al. reported a molecularly imprinted fluorescence probe (B-CQDs@Eu/MIPs) to detect the ratio fluorescence of tetracycline by combining B-CQDs derived from passion fruit peels and Eu^3+^ using microwave pyrolysis and sol-gel methods [62]. SEM and TEM results showed that the modification of Eu^3+^ and MIPs on the surface of B-CQDs could change its shape and size. With the addition of Eu^3+^, the transition of some electrons from the excited state to the ground state in B-CQDs was blocked by tetracycline, resulting in the reduction of its blue fluorescence emission at 457 nm, while the complexation of tetracycline and Eu^3+^ led to the enhancement of red fluorescence intensity of B-CQDs at 620 nm due to the antenna effect. The tetracycline concentration showed a good linear relationship in the range of 25–2000 nM, with the LOD of approximately 7.9 nM. RSD values (1.5–5.3%) and the recovery rate of 94.2–103.7% in milk samples, which suggested that the ratio fluorescence probe B-CQDs@Eu/MIPs with good precision and practicability could be applied to monitor tetracycline in food safety and water environment. However, the synthesis route of B-CQDs@Eu/MIPs was relatively tedious and needed to be further simplified to accommodate its large-scale production.

Beena Mathew et al. synthesized the CDs to simultaneously detect fluoroquinolone and tetracycline antibiotics using Curcuma amada as carbon source for the first time [63]. When tetracycline was added, the fluorescence intensity of the synthesized CDs was significantly reduced due to inner filter effect, but was enhanced in the presence of fluoroquinolone as a result of hydrogen bonding. Based on the relationship between fluorescence response of CDs and tetracycline concentrations (0–16 μM), an LOD was calculated to be 33 nM. In addition, the synthesized CDs had the same linear detection concentrations, and the LOD for fluoroquinolone was 2 nM. Moreover, they could serve as an electrochemical probe for tetracycline identification with an LOD of 0.5 nM. The CDs sensor provided two trusted modes for antibiotics detection in the environmental analysis. However, these two antibiotics worked at very narrow pH values, which did not contribute to the analysis in real samples.

Although thiamphenicol (TAP) can treat some infections and diseases, its residue can cause various symptoms, such as hypersensitivity, ulcers and kidney necrosis [64]. A down/up-conversion multi-emission ratiometric FL-MIPs sensor bCDs@SiO2@aCDs@ZIF-8@MIP was first reported by Zhang Zhaohui et al. for visible fluorescence sensing of TAP [65]. The biomass CDs (bCDs) was synthesized with fresh coriander as starting material. The purpose of introducing ratiometric FL-MIPs into this sensor was to enable the self-calibration and visualization of changes in fluorescence color. Embedding metal-organic frameworks (MOFs) in this sensor could not only produce the signal amplification effect of fluorescence sensing, but also prevent the self-quenching of CDs. TEM results showed that the sensor has an average size of 250 nm, with a significant core-shell structure covering an imprinted layer of approximately 50 nm. After TAP was added, the fluorescence of this sensor was quenched, accompanied by a visible fluorescence color changing from blue to purple to red under UV irradiation at 365 nm due to the photoinduced electron transfer effect. Under different excitation wavelengths (370 nm and 780 nm), TAP concentration detected by this sensor displayed two linear ranges (5.0 nM–6.0 μM and 6.0–26.0 μM) with an LOD of 1.9 nM and 0.9 nM, respectively. Although this sensor had achieved satisfactory results in the detection of TAP, its synthetic process was complicated and difficult to prepare on a large scale.

Ascorbic acid is an important micronutrient that plays an important role in biological systems, including as antioxidant, co-factor of enzymatic reaction, free-radical scavenger, cell division, etc. [66]. However, long-term excess ascorbic acid can cause diarrhea, nausea, stomach cramps and other symptoms. Using grape seeds and thiourea as starting materials, Zhu Xiashi et al. prepared an on-off-on fluorescence probe (N, S-CDs) to simultaneously detect Cu^2+^ and ascorbic acid [67]. The surface of N, S-CDs was rich in nitrogen and sulfur functional groups according to the results of FTIR and XPS. After the addition of Cu^2+^, static quenching mechanism led to the decrease in fluorescence intensity of N, S-CDs and then with the addition of ascorbic acid, the fluorescence emission of N, S-CDs/Cu^2+^ mixed system was restored because ascorbic acid and Cu^2+^ were complexed, separating from the surface of N, S-CDs. There was a good linearity in the concentration range between Cu^2+^ (150–500 µg/mL) and ascorbic acid (0.1–400 µg/mL), together with a low LOD of 0.048 mg/L and 0.036 mg/L for Cu^2+^ and ascorbic acid, respectively. MTT assay showed a low cytotoxicity of N, S-CDs to human DU145 cells, providing a feasible path for cell imaging and in vivo detection. Carbon quantum dots (CQDs) for photocatalysis and detection of Fe^3+^ and ascorbic acid was developed by TaeYoung Kim et al. with pear juice via the solvothermal process [68]. With the addition of Fe^3+^, the fluorescence intensity of CQDs was quenched and then after ascorbic acid was subsequently added, the fluorescence emission of the mixed system CQDs/Fe^3+^ was released. The LOD for Fe^3+^ and ascorbic acid sensing were 2.28 µM and 1.27 µM, respectively. More interestingly, the CQDs exhibited superior efficient visible-induced photocatalytic activity, where dye methylene blue (MB) was almost completely degraded (99.5%) within 130 min under visible-light irradiation as a result of their efficient light absorption, electron transfer and separation of photogenerated charge carriers. At that time, carbon dots capable of biodegrading dyes and simultaneously detecting Fe^3+^ and ascorbic acid was first reported, opening up the possibility of building multifunctional devices. With the help of the quenching effect between Cu^2+^ or Fe^3+^ and CDs, the selective detection of ascorbic acid was achieved, which, however, was likely to cause interference and make an operation more complex.

**Figure 10:**
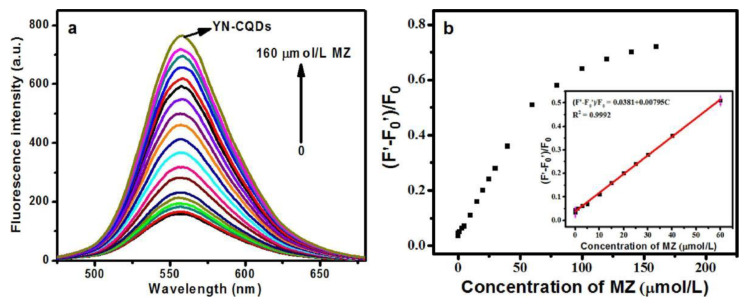
Schematic illustration of (**a**) fluorescence emission spectra of the YN-CQDs/Ag^+^ system upon addition of various concentrations of MZ. (**b**) relationship between recovery efficiency and the concentration of MZ. Inset: a linear relationship of recovery efficiency versus the concentration of MZ over the range from 0.003 to 60 μM. Adapted with permission from Yu et al., 2021, Copyright 2021. Reproduced with permission from Elsevier [70].

Methimazole can treat human hyperthyroidism, but excessive intake can cause adverse reactions, such as arthralgia, rare nephritis and liver cirrhosis in human bodies [69]. Therefore, it is of great importance to efficiently and sensitively detect methimazole in clinical chemistry and pharmaceutical fields. Deng Biyang et al. prepared a bright yellow fluorescent nitrogen-doped carbon quantum dots (YN-CQDs) with lettuce leaf as the starting material through hydrothermal technique to monitor methimazole [70]. The quantum yield of YN-CQDs was as high as 38% and the fluorescence intensity of YN-CQDs remained basically unchanged after being stored for 2.5 months, indicating that YN-CQDs had better optical physical properties than other biomass-based carbon dots reported. After the addition of Ag^+^, the fluorescence emission of YN-CQDs decreased significantly owing to static quenching, but with the addition of methimazole to the mixed system YN-CQDs/Ag^+^, the YN-CQDs emitted fluorescence emission again because methimazole had a higher affinity for Ag^+^ and Ag^+^ escaped the YN-CQDs surface. The average size of YN-CQDs/Ag^+^ was 459 nm, larger than that of YN-CQDs (91.3 nm), and after the addition of methimazole, the average size of YN-CQDs was roughly recovered to 122 nm, proving that methimazole could form a complex Ag^+^/methimazole with Ag^+^, which had also been verified by zeta potential tests. The YN-CQDs/Ag^+^ sensor presented a linear methimazole concentration range (0.003–60 μM) and a calculated LOD of 0.8 nM (Figure 10). The satisfactory recoveries (99.00–102.0%) and RSDs (lower than 3.2%) of methimazole detected in mouse plasma samples provided a convenient technique for methimazole determination in practical applications. Water was generally used as the solvent in the application of traditional hydrothermal method, but ethanol and acetone were used for preparing YN-CQDs in this literature, which would cause serious environmental pollution if YN-CQDs were produced in large scale.

Nitroimidazole antibiotics (5-NDZs) can treat infections caused by bacteria or protists. However, 5-NDZs can accumulate in human bodies and induce neuritis, immune hepatitis, shock and other diseases if excessively used [71]. Niu Na et al. reported a fluorescence sensor CDs@MIPs based on catalpa walnut shells-derived CDs and molecularly imprinted polymers (MIPs) for accurately identifying 5-nitroimidazole antibiotics (5-NDZs) through hydrothermal and sol-gel methods [72]. With the addition of 5-NDZs, the fluorescence intensity of CDs@MIPs decreased significantly, which was attributed to the formation of a ground-state complex, resulting in static quenching. A wide linear working range of CDs@MIPs towards 5-NDZs concentrations (20–5000 nM) was achieved. CDs@MIPs with 100% identification accuracy for 5-NDZs in buffer solution and spiked water samples indicated that this sensor array provided a very reliable tool for environmental analysis. Compared with other sensors for detecting 5-nitroimidazole, CDs@MIPs had a narrow linear working range, which would also limit its wide applications.

Alizarin red S (ARS) is an organic dye widely used in calcium staining of tissue slices in biological studies. Excessive discharge of ARS will cause water pollution, so it is of great significance to efficiently detect ARS [73]. Cui Xuejun et al. Developed a carbon quantum dots (CQDs) to sensitively detect Alizarin red S (ARS) with corn stalk shell by hydrothermal approach [74]. After ARS was added, the fluorescence emission of CQDs was quenched due to the combination of CQDs and ARS. The fluorescence intensity of CQDs had a linear relationship with the concentration of ARS in the range of 10–150 µM, and the detection limit was 2.65 µM. After incubating in the CQDs solution (100 mg/L) for 48 h, the cell viability of A549 cell still exceeded 75%, indicating that the CQDs had a good biocompatibility and thus could be used for bioimaging and in vivo monitoring. The use of near-critical water as the reaction solvent not only facilitated the partial dissolution of lignocellulose but also contributed to the preparation of CQDs by using it as an oxidant and catalyst.

The surfactant residue in laundry powder has become an important environmental problem, which poses a serious threat to microalgae, macrophytes and fish [75]. Therefore, it is of great significance to develop novel biomass-based carbon dots to realize efficient detection of laundry powder. Zhao Longshan et al. synthesized carbon quantum dots (CQDs) to detect laundry powder with chicken bones as carbon source [76]. With addition of sodium dodecyl benzene sulphonate of a major anionic surfactant in laundry powder, the fluorescence intensity of CQDs was significantly enhanced, but it was not the same case after the addition of other substances, such as sodium dodecyl sulfate (SDS), NP40 and Vitamin c. However, the ingredients in laundry powder are rather complicated, which may interfere with the detection process of CQDs.

## 3. Physical Sensors

Temperature affects almost all physical changes and chemical reactions and is very vital to the real-time monitoring of human activities, plant and animal growth and various industrial processes [77]. Traditional temperature measurement technologies, such as volumetric expansion thermometer, infrared thermometer, etc., are generally faced with the defects that they can only measure the surface temperature of objects and are susceptible to the interference of strong electric and magnetic fields.

**Figure 11:**
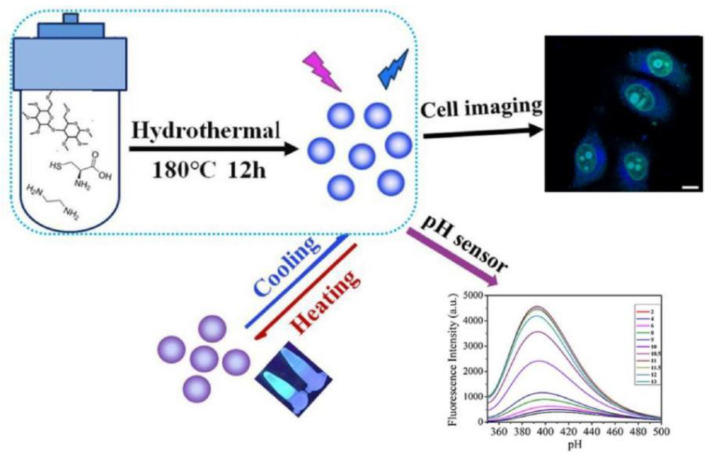
Schematic illustration of the synthetic procedure of N, S-CDs and multifunctional applications of pH detection, temperature monitoring, RNA-selective imaging. Adapted with permission fromLiu et al., 2021, copyright 2021. Reproduced with permission from Elsevier [78].

Using methyl cellulose, Yu Xiaoqi et al. prepared L-cysteine and ethylenediamine as raw materials, a nitrogen and sulfur co-doped carbon dots (N, S-CDs) through hydrothermal method to simultaneously detect pH and temperature [78]. TEM results showed that nitrogen and sulfur elements were doped into the N, S-CDs skeleton to interact with other atoms on the N, S-CDs surface, which affected the sizes of N, S-CDs. With the increase of temperature, the fluorescence intensity of N, S-CDs decreased gradually, which was linearly related to temperature (10–70 °C) due to the surface defect of N, S-CDs. However, at the temperature higher than 70 °C, most excited electrons returned to the ground state in a non-radiative manner, resulting in a reduction of fluorescence intensity of N, S-CDs. When pH value was between 2 and 6, the fluorescence intensity of the N, S-CDs remained stable. As pH increased from 6 to 9, the PL intensity began to increase slowly, while that of the N, S-CDs was enhanced significantly in the pH range of 9–12. There was an excellent linear correlation between pH (10–11.5) and the PL intensity of N, S-CDs, suggesting that N, S-CDs could work as a pH indicator. The cell viability of HepG2 cells was still 71.3% after being incubated in N, S-CDs (1 mg/mL). Meanwhile, the N, S-CDs was distributed not only in the cytoplasm but also in the nuclear region as densest spots and presented a bright blue fluorescence signal under radiation excitation at 405 nm, indicating that low-toxicity N, S-CDs was suitable for living cells imaging and also demonstrating intrinsic nucleus location capability. As described in Figure 11, N, S-CDs showed a great potential for multifunctional applications in pH detection, temperature monitoring, RNA-selective imaging and other fields. The fluorescence intensity of N, S-CDs exhibited a wide linear temperature response in the range from 10 to 70 °C, which was suitable for many temperature scenarios. However, very narrow linear pH response (10–11.5) was not helpful in acidic and alkaline environments.

Three N-doped carbon dots (N-CDs) of N-cCDs, N-lCDs and N-xCDs were synthesized by Chen Congjin et al. with cellulose, lignin and xyloses as carbon sources through hydrothermal method, respectively [79]. After hydrothermal treatment, some agglomerated CDs were also observed in the reaction residue of cellulose, and some small particles were left through xylose hydrothermal treatment, but lignin was combined into thick flakes during treatment, which was responsible for the low quantum yield of N-lCD. There was a good linear relationship between the FL quenching intensity of N-cCDs and N-xCDs and temperature (9–80 °C), and the fluorescence intensity of N-lCDs remained almost constant in the temperature ranges of 9–30 °C and 35–80 °C and increased dramatically in the temperature range of 30–35 °C. N-cCDs and N-xCDs showed excellent performance in sensing temperature, while N-lCDs did not. Under the excitation of 365 nm UV lamp, both N-cCDs and N-xCDs emitted bright white fluorescence, and N-lCDs presented weak fluorescence as a result of low quantum yield. N-cCDs and N-xCDs solution presented fluorescence under 254 nm UV irradiation when directly written on silk and cotton, while eiderdown did not show fluorescence and thus was identified. Silk displayed bright fluorescence under 365 nm UV light, while cotton and eiderdown did not. The fluorescence of N-cCDs, N-lCDs and N-xCDs was quenched by Hg^2+^ ions but not by other metal ions. According to the fluorescence lifetime tests, the quenching mechanism of Hg^2+^ on N-cCDs and N-xCDs was possibly attributed to the formation of non-fluorescent complexes between Hg^2+^ and the surface functional groups of N-cCDs and N-xCDs, and the fluorescence quenching between Hg^2+^ and N-lCDs may be caused by fast electron transfer process. These results suggested that the luminescence process of N-cCDs and N-xCDs was different from that of N-lCDs. Therefore, biomass-based CDs can be used for a variety of applications, including temperature, Hg^2+^ sensing and fluorescent ink. The very high content of lignin in biomass may have a great impact on the sensing performances of the CDs. So far, biomass-based CDs sensors for pH, temperature and pressure sensing have been rarely reported.

## 4. Biosensors

**Figure 12:**
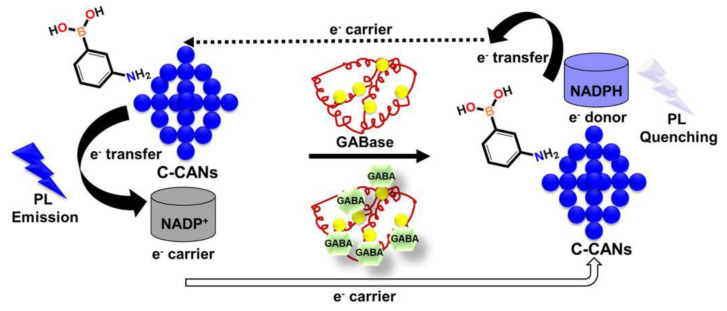
Schematic illustration of possible PL quenching mechanism for the detection of GABA using C-CANs with GABase. Adapted with permission from Sangubotla et al., 2022, copyright 2022. Reproduced with permission from Elsevier [81].

As the abnormal level of γ-aminobutyric acid (GABA) may be related to neurodegenerative diseases, it is necessary to exploit simple and feasible carbon dots sensors for its efficient detection [80]. Jongsung Kim constructed an enzyme-based fluorescent biosensor C-CANs for detecting GABA and modified the carbon dots derived from coffee waste with 3-aminophenylboric acid (APBA) and nicotinamide adenine dinucleotide phosphate (NADP^+^) [81]. After GABA was added, the fluorescence quenching of C-CANs was triggered by GABase enzyme due to electron transfer (Figure 12). The linear concentrations (0–20 µM) of GABA detection were obtained, with the LOD of 95.09 nM. Meanwhile, this sensing platform demonstrated an excellent potential for the detection of GABA in human serum samples and intracellular. However, its preparation and detection processes were complicated, which was not conducive to widespread use.

Glucose is an indispensable nutrient in the metabolism of human bodies. Glucose will lead to a variety of diseases, such as pallor, palpitations, diabetes, etc. if its concentration is too low or too high [82]. Zhao Longshan et al. synthesized chicken cartilage-based carbon dots (cc-CDs), which could work as a glucose sensor using H_2_O_2_ as a bridge [83]. The sensing process was briefly described as follows: glucose was catalyzed by glucose oxidase (GOx) to produce H_2_O_2_, which then reacted with Fe^2+^ to generate Fe^3+^ and ▪OH, leading to the decrease of the fluorescence intensity of cc-CDs as a result of the dual actions of Fe^3+^ and ▪OH. A good linear relationship between the fluorescent response of cc-CDs and glucose concentration (5–1000 μM) was obtained with an LOD of 1.22 μM. Meanwhile, the synthesized cc-CDs also demonstrated low cytotoxicity and thus was applicable in actual serum samples. However, prior to glucose measurement in real serum samples, the samples must be properly diluted to reduce the interference of endogenous ascorbic acid and glutathione contents on measurement results.

Glutathione (GSH) plays an important role in a series of biochemical processes, such as maintaining cellular REDOX activity, metabolism and gene regulation [84]. Abnormal levels of GSH have been linked to many types of cancer, Parkinson’s disease, liver damage, cardiovascular disease and childhood autism, which makes it important to exploit novel CDs for real-time detection of GSH [85]. Shi Qingshan et al. designed a long-wavelength rose-red fluorescence emission (654 nm) carbon dots (wCDs) to detect glutathione (GSH) in two interesting ways using biomass Wedelia trilobata as raw material through microwave-solvothermal technique [86]. Based on the dynamic quenching effect, the fluorescence intensity of wCDs at 654 nm was quenched by Cu^2+^, and there was a good linear relationship between Cu^2+^ concentration (0.1–20 µM) and fluorescence quenching efficiency of wCDs with an LOD of 0.22 µM. When GSH ([GSH] < [Cu^2+^]) was introduced into the wCDs-Cu^2+^ mixed system, the quenched fluorescence of wCDs was not completely recovered caused by the blocked interaction between wCDs and Cu^2+^. Unfortunately, with the continuous increase of GSH concentration ([GSH] > [Cu^2+^]), the restored fluorescence of wCDs was quenched again due to the interaction between wCDs and GSH. With the increase of GSH concentration (0–20 mM), the fluorescence intensity of wCDs at 654 nm gradually decreased, and the emission peak of wCDs was shifted from 654 nm to 641 nm, which may be caused by the interaction between wCDs and GSH to produce a new radiation recombination center. Under 365 nm UV light, GSH quenched the fluorescence intensity of wCDs within 20 s. The wCDs presented a good linear equation for GSH (0–3.0 mM) with an LOD of 35 μM, which was consistent with the GSH content in living cells (0.5–10 mM). Due to low toxicity, advantageous biocompatibility and excellent permeability, wCDs can not only detect Cu^2+^ and GSH in cells, but also distinguish cancerous cells from normal ones. The literature on the direct and indirect detection methods of GSH by CDs was extremely limited. The relationship between Cu^2+^ and GSH concentrations could not be easily balanced, which made practical applications more difficult.

Cytochrome c plays an important role in mitochondrial respiratory chain and cell apoptosis [87]. Abnormal trypsin levels can lead to a variety of pancreatic symptoms, such as pancreatitis, cystic fibrosis, etc. [88]. Niu Na et al. synthesized biomass nitrogen-doped carbon quantum dots (N-CQDs) for detecting cytochrome c and trypsin with cellulolytic enzyme lignin as carbon precursor and ammonia as nitrogen precursor via hydrothermal technology [89]. As the concentration of cytochrome c increased, the fluorescence intensity of N-CQDs gradually decreased, which was induced by electrostatic induction aggregation and static quenching according to the results of zeta potential, TEM, fluorescence lifetime and absorption spectra. After the addition of trypsin, the fluorescence emission of N-CQDs/cytochrome c mixed system was gradually restored, because cytochrome c was hydrolyzed by trypsin into peptide fragments, resulting in the disappearance of aggregation induced quenching. In addition, a good linear relationship between the fluorescence response and cytochrome c (1–50 μM) and trypsin concentrations (0.09–5.4 U/mL) was obtained, with an LOD of 0.29 μM and 0.013 U/mL, respectively. The recoveries and RSD values in serum samples proved that the biomass N-CQDs provided a new approach for clinical diagnosis. However, the CQDs prepared from biomass may contain impurities, which will affect the trypsin activity and thus change the experimental results.

MNase, a nonspecific endonuclease, has been recognized as a threat agent due to its ability to digest double and single stranded DNA, causing many infections and diseases, such as rash, diarrhea, nausea, etc. [90]. R. Geetha Balakrishna et al. reported a probe C-dots-dsDNA-GO for the detection of micrococcal nuclease (MNase) using single stranded DNA (ssDNA) conjugated C-dots (derived from maize biomass) and graphene oxide (GO) [91]. ssDNA-C-dots interacted with ssDNA-modified GO to form a DNA double bridge, resulting in energy transfer from C-dots to GO, and finally quenching the fluorescence of C-dots. The energy transfer interaction between C-dots and GO was inhibited by using the properties of MNase to cleave DNA, restoring the fluorescence emission of C-dots and realizing the detection of MNase. The C-dots-dsDNA-GO sensor had successfully determined MNase in a wide linear range of 0.0004–0.2 U/mL with an LOD of 0.002 U/mL. The low-cytotoxicity C-dots can work as a potential biosensor for recognizing pathogens and their analogues. The preparation process of the probe C-dots-dsDNA-GO and its detection of MNase was relatively complicated, posing a great challenge to untrained technicians.

*E. coli* often causes serious health problems, such as meningitis, anemia and bloody diarrhea, so the use of novel biomass cd sensors for the rapid and sensitive detection of *E. coli* is critical for environmental science, food safety and clinical diagnosis [92]. Utilizing raw potato as biomass material, Amrita Chatterjee et al. exploited a novel carbon dots (CDs)-manganese dioxide (MnO_2_) nanosheets fluorescence probe to monitor Escherichia coli (*E. coli*) by enzymatic redox reaction [93]. The electrostatic interaction between MnO_2_ nanosheet and CDs led to the fluorescence quenching of CDs. The enzyme in the respiratory pathway of *E. coli* converted p-benzoquinone (BQ) into hydroquinone (HQ) by two electron reduction, which then reacted with MnO_2_ nanosheet to produce Mn^2+^ ions. As MnO_2_ was decomposed, CDs were released from electrostatic interaction and became dispersed again, thus emitting blue fluorescence emission (445 nm). The probe exhibited a highly selective and sensitive detection performance on *E. coli*, with a linear working range of 50–1.0 × 10^9^ CFU/mL and an LOD of 50 CFU/mL. In addition, the sensing platform enabled the quantitative analysis of *E. coli* in rainwater and food samples. However, p-benzoquinone and hydroquinone were used in the detection process, which were more likely to cause secondary pollution to the environment.

As we all know, bacteria are easy to cause diseases, such as cold, diarrhea, fever, etc. Hence, it is urgent to develop efficient fluorescent CDs for bacteria detection. Liu Yu et al. developed an N-C dots probe with bacterial cellulose and urea as raw materials through hydrothermal carbonization method [94]. AFM and TEM datum showed that N-C dots were quasi-spherical and featured more uniform and smaller size distribution (1.0–2.0 nm), resulting in stronger blue fluorescence emission. Surprisingly, yeast cells labeled by fluorescent showed different fluorescence effects in the visual field, while the cells with N-C dots displayed slightly stronger fluorescence effects under Rhod filters. Therefore, N-C dots can be used as a potential fluorescent probe for cell imaging and can be further used as a sensitizer for bacterial detection.

## 5. Discussion and Conclusions

In this article, we summarized the preparation processes, properties and sensing applications of biomass-based CDs. By utilizing different biomass as carbon sources, biomass-based CDs were functionalized to construct chemical physical and biological sensors by employing various synthetic techniques, including hydrothermal method, thermal carbonization, ultrasonic-assisted wet-chemical-oxidation process, acid-treatment-assisted pyrolysis as well as microwave heating. However, most of the synthetic methods require high temperatures and strong acids and bases, and it is urgent to develop new technologies that are mild, low-cost and environmentally friendly. The biomass-based CDs fabricated with plant and animal derivatives and industrial organic wastes as carbon sources exhibit excellent sensing properties against chemical components, physical factors and biological compositions. By making biomass resources into CDs, not only is waste effectively utilized but also environmental pollution caused by random discharge is avoided. According to the experimental results of fluorescence, UV and lifetime, the mechanisms of fluorescence quenching or enhancement of biomass-based CDs may be as static quenching, dynamic quenching, photoinduced electron transfer (PET), förster resonance energy transfer (FRET) and inner filter effect (IFE). The static quenching and dynamic quenching may co-occur in the same biomass-based CDs. PET mechanism is expressed as: CDs do not emit or emit very weak fluorescence before binding to the analyte, which is relatively rare for biomass-based CDs. FRET mechanism is more common for biomass-based CDs. IFE mechanism can improve the sensitivity by converting the absorbed signal into a fluorescent signal. Compared with simple quenching or enhancement mechanism, new ratiometric probe developed based on CDs can eliminate the background interference caused by changes in the external environment and the abnormal change of correction signal, thus improving the detection sensitivity.

Although biomass-based CDs have demonstrated high efficiency and superior sensing abilities, there are still some challenges to be overcome in order to improve the practical applications in the future: (1) The yield of biomass-based CDs is still very low in the laboratory stage, far from meeting the requirements of large-scale preparation and industrial production. (2) The surface composition and structure of biomass-based CDs are ambiguities, which may lead to relatively low quantum yield and imperfect properties. Hence, it is urgent to develop a controllable strategy of “design-preparation-application”. (3) The fluorescence of most biomass-based CDs in strong acidic or alkaline medium is usually unstable, thus limiting their practical applications in environmental analysis. (4) The fluorescence and sensing mechanisms of biomass-based CDs remain unclear. (5) Few biomass-based CDs are able to achieve simultaneous multi-functional detection, which still need further research and exploration. A large number of biomass-based CDs sensors have been developed very few of which, however, can be commercialized. With our continuous efforts, commercially available multifunctional biomass-based CDs will certainly be developed. We sincerely hope that this article will provide inspiration for researchers to develop better biomass-based CDs sensors.

## Data Availability

No applicable.
